# A Prototype Process for Demulsification of Waste Ice Cream

**DOI:** 10.1002/fsn3.4626

**Published:** 2024-12-12

**Authors:** Rafael A. Garcia, Chen Liang, Benjamin M. Plumier, Changhoon Lee, Lorelie P. Bumanlag, John A. Renye, Peggy M. Tomasula

**Affiliations:** ^1^ Dairy and Functional Foods Research Unit, Eastern Regional Research Center, Agricultural Research Service U.S. Department of Agriculture Wyndmoor Pennsylvania USA

**Keywords:** butterfat, dairy product, de‐emulsification, fat quality, food loss, ice cream, protease

## Abstract

Recovery of the butterfat in waste ice cream may be an opportunity to mitigate food and economic loss. Previous efforts to recover such fat have succeeded in producing a fat‐enriched fraction but have not succeeded in demulsifying the fat. In the present study, a method involving a sequence of emulsion‐breaking steps is shown to be effective for releasing a majority of the fat from waste ice cream as free, unemulsified oil. The effect of altering process conditions including enzyme type, pH, and incubation temperature is reported. Depending on the test conditions and the variety of ice cream used, typically 59%–81% of the fat was recovered, with varying degrees of hydrolytic and oxidative damage. As the method is relatively complex, an experiment which omitted individual processing steps demonstrated that each step was required for high recovery. Success with 4 of 5 tested varieties showed that the method has reasonably broad applicability. The results are compared with those achieved using a standardized solvent extraction method. Finally, the method is evaluated for its potential as the basis for a commercial WIC fat recovery process.

## Graphical abstract

Recovery of the butterfat in waste ice cream may be an opportunity to mitigate food and economic loss, but previous efforts have not succeeded in demulsifying the fat. In the present study, a method involving a sequence of emulsion‐breaking steps is shown to be effective for releasing a majority of the fat from waste ice cream as free, unemulsified oil. The method described is better adapted to commercial processing than the only other published method.

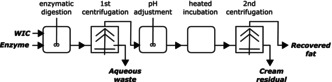

## 1 Introduction

Ice cream manufacture results in a considerable volume of product that is edible but will nevertheless be wasted rather than being reworked or sent to retail outlets. The particular causes for this waste are too numerous to detail here, but many are linked to the perishability of ice cream mix, the inability to pause the operation of a continuous ice cream freezer, low tolerance for product quality defects, or allergen contamination concerns (Garcia et al. 2023). Recapture of food and economic value from this waste ice cream (WIC) has been identified as a priority issue by this industry (Anonymous 2019).

Reuse of WIC in human food is greatly complicated by allergens. Ice cream manufacturers use many of the most common food allergens (dairy, egg, wheat, soy, peanut, tree nut) within a single facility (Goff and Hartel 2013a), and the systems that exist to prevent cross‐contamination in production cannot practically be applied to WIC. Commercial food processing is typically not capable of removing or destroying allergens, with some exceptions. In the US, highly refined oils are exempt from food allergen regulations (Public Law 108–282, Title II—Food Allergen Labeling and Consumer Protection Act of 2004), and research shows that such oils can be safely consumed by sensitive consumers (Crevel, Kerkhoff, and Koning 2000; Ramazzotti et al. 2008). Consequently, it may be possible to recover butterfat from WIC and refine it such that it can be used in food products without restriction.

A process capable of transforming WIC into refined butterfat will require an initial stage that concentrates crude fat. A laboratory standard for recovering fat from ice cream exists (Anonymous 2012), but it involves the addition of large proportions of solvent, salt, and water, evaporation of the solvents, and disposal of the aqueous and salt byproducts. For industrial application, a nonsolvent extraction method may be preferrable, even if there are tradeoffs in fat yield and quality.

The fat in WIC is emulsified and the emulsion is stable enough that free fat will not spontaneously separate from liquid WIC. Some WIC, however, creams in a manner that superficially resembles the creaming of unhomogenized whole milk; an upper, fat‐enriched layer slowly develops. Such creaming can be promoted by heat and centrifugation but the fat remains emulsified (Garcia et al. 2023). Treatment with protease enzymes has been shown to similarly promote creaming without demulsification; in a study involving four different industrial proteases, the investigators found a positive correlation between degree of protein hydrolysis and creaming rate (Liang et al. 2023). The cream described in this section is not a satisfactory input for conventional refining processes which typically handle nonemulsified (but impure) oil.

The previous work has employed many of the most common emulsion breaking strategies which include applying heat, pH adjustment, destruction of the emulsifying agent, and centrifugation (Lee 1983; Rajvaidya and Markandey 2005). In informal and exploratory research in our laboratory, it was found that a specific sequence of these treatments (described in Section 2.2) yielded a continuous phase of free oil. The purpose of the present study is to examine the performance of this experimental fat separation method in terms of the proportion of fat recovered and the hydrolytic and oxidative damage to the fat. Key parameters of the method, including enzyme type, pH, and incubation temperature, are varied one‐at‐a‐time for the purpose of improving the method. The method is examined to determine whether it is unnecessarily complex by omitting individual processing steps. Finally, the method is evaluated for its potential as the basis for a commercial WIC fat recovery process.

## 2 Materials and Methods

### 2.1 Materials

Five varieties of vanilla ice cream were purchased from local supermarkets and distributors and used within 6 months of purchase. The commercial enzyme preparations *Formea CTL* (300 KPROT/g; Novozymes, Bagsværd, Denmark) and *Rennet for Cheese Making* (20,000 U/g, Creative Enzymes, Shirley, NY) were stored according to the manufacturer's recommendations. All chemicals were reagent grade or better.

### 2.2 Fat Separation Methods

Fat was separated from ice cream by either a standard method (AOAC 960.32; Anonymous 2012) or by the experimental method that is the primary subject of this study. The standard method was scaled down by half to conserve reagents. Briefly, it involved dilution of melted ice cream with water, addition of ammonium hydroxide, and shaking in a separatory funnel with successive additions of ethanol, diethyl ether, and petroleum ether. After draining the aqueous layer, the upper layer was dried using Na_2_SO_4_ and filtered. Solvent was allowed to evaporate from the filtrate overnight at 55°C, and the remainder was weighed at room temperature. Each standard separation was replicated three times.

**FIGURE 1:**
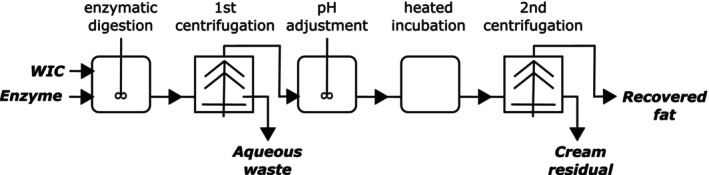
Schematic representation of experimental fat separation method.

The experimental fat separation method comprised main five steps—enzymatic digestion, 1st centrifugation, pH adjustment, hot incubation, and 2nd centrifugation (Figure 1). In more detail, ice cream was portioned into flasks while still frozen and then equilibrated to 40°C before adding enzyme at a dose of 1% w/w enzyme/ice cream protein; enzymatic digestion proceeded with 100 rpm shaking at 40°C for 1 h. Digestate was then treated in a centrifuge that had been preheated to 40°C, in a swinging bucket rotor at 4000 × *g* for 10 min. The bottom layer was removed by vacuum and discarded. pH of the remaining material was adjusted to the value prescribed for the experiment (±0.2 units) using 3 N NaOH. The material was then incubated in a circulating water bath at the temperature prescribed for the experiment for 30 min. Finally, the material was centrifuged again, using the same conditions. Mass of oil recovered was determined indirectly by measuring the depth of oil in the centrifuge tube, converting to volume using a depth–volume correlation, and then using the specific gravity to convert to mass. Specific gravity was taken as:

(1) Specific gravity of butterfat=0.8974−0.00068×∆T

where Δ*T* is the difference between incubation temperature and 48.9°C (Bailey 1919; Paquot 1979). Each experimental separation was replicated 3–4 times.

### 2.3 Analytical Methods

The fat content of whole ice cream and recovered fat were determined in triplicate by time domain NMR (ORACLE Rapid NMR Fat Analyzer, CEM Corp., Charlotte, NC, USA). This involved drying samples onto glass fiber pads, wrapping in a proprietary film, and equilibrating to 40°C, prior to analysis.

Free fatty acids (FFA) and peroxide value (PV) were determined in duplicate using FoodLab Junior (CDR s.l.r., Florence, Italy). Analyses were conducted immediately after fat separation. The results for FFA are expressed as a percentage of oleic acid, while PV is expressed as milliequivalents of oxygen per kilogram (meq O_2_/kg).

## 3 Results and Discussion

### 3.1 Characteristics of Ice Cream Varieties Studied

The ice cream varieties included in the study set (Table 1) are all similar in that they are commercial vanilla products with no inclusions other than, in some cases, vanilla bean flakes; all had fat contents in the range 9.7%–10.5%. The varieties were diverse in other respects; each had a different system of added emulsifiers, and they differed in the extent to which they cream passively.

**TABLE 1:** Characteristics of ice cream varieties studied.

Variety ID	Brand	Product name	Emulsifiers^a^	Fat (%, wb)^b^	Passive creaming^c^
P V	Potts	Vanilla	MDG + Poly 80	10.0 ± 0.6	Low
TH VB	Turkey Hill	Vanilla Bean	MDG	10.2 ± 0.1	None
B NV	Breyers	Natural Vanilla	None	9.7 ± 0.1	Very high
TH FV	Turkey Hill	French Vanilla	MDG + egg yolk	10.5 ± < 0.05	None
HB V	Homemade Brand	Vanilla	Egg yolk	10.5 ± 0.1	None

^a^ MDG, mono‐ and diglycerides of fatty acids; Poly 80, polysorbate 80.

^b^ wb, wet basis; values are means of three replicates ±1 standard deviation.

^c^ From: Garcia et al. (2023). This reference also contains further data on the composition of these varieties.

### 3.2 Standard Fat Separation Method

The standard separation method recovered a very high proportion of the total fat from each variety tested (Table 2). Analysis of the recovered fat in the ORACLE fat analyzer returned results of 100% in each case (data not shown); these results are taken only as evidence confirming that the recovered material is mostly fat and not as a measure of purity.

**TABLE 2:** Fat recovery and quality using the standard fat separation method. Values are means of three replications ±1 standard deviation.

Variety ID	Fat recovery	FFA^a^	PV^b^
	%	% as oleic acid	meq O_2_/kg
P V	102.3 ± 7.3	0.06 ± 0.01	1.57 ± 0.26
TH VB	97.6 ± 0.8	0.04 ± 0.01	0.76 ± 0.16
B NV	101.9 ± 1.9	0.05 ± 0.02	0.82 ± 0.05
TH FV	102.5 ± 0.8	0.06 ± 0.02	0.34 ± 0.03
HB V	99.2 ± 1.1	0.03 ± 0.01	0.27 ± 0.02

^a^ Free fatty acids.

^b^ Peroxide value (milliequivalents O_2_/kg).

The quality of the recovered fat was relatively high. The level of free fatty acids (FFA) is commonly used as a primary indicator of fat quality. Free fatty acids are a product of the hydrolysis of triacylglycerols; free fatty acids are undesirable in edible oils because they promote fat oxidation (Frega, Mozzon, and Lercker 1999) and, at sufficient concentration, cause perceptible rancidity (Wiking 2011). The FFA results are close to a voluntary industry standard maximum FFA of 0.05% for newly refined oil (Dunford 2016).

The peroxide value (PV) is another commonly used indicator of fat quality. PV describes the level of hydroperoxides present; hydroperoxides are formed in the initial stage of edible oil oxidation (Dunford 2016). Refined oils usually have PV of < 1 meq/kg; oils are considered oxidized with PV > 3 meq/kg (Dunford 2016). None of the varieties produced oxidized fat by this criterion. Although the separation method involves substantial shaking in an air‐filled separatory funnel, any oxidation that occurs during these steps seems to be below the level of practical significance.

While the technical performance of the standard separation method was very good, it consumed large volumes of reagents and generated large volumes of waste. An extraction yielding ~10 g recovered fat consumed 100 mL ethanol, 100 mL diethyl ether, 100 mL petroleum ether, 50 mL water, 25 mL ammonium hydroxide, and 13 g sodium sulfate. It produced both aqueous and organic waste streams; although in an industrial implementation evaporated organic waste would presumably be recaptured, in this research the polluting gases were vented to the atmosphere.

### 3.3 Experimental Fat Separation Method—Enzyme Type

Two industrial protease preparations were included in this study of the experimental fat separation method; as proprietary formulations, some of their characteristics are not revealed by the manufacturer. Formea CTL was chosen because in a previous study comparing four industrial proteases (Liang et al. 2023), it was a relatively strong promoter of WIC creaming. The manufacturer describes it as a “chymotrypsin‐like microbial endo‐protease,” and presumably, like chymotrypsin it is relatively nonspecific and is capable of hydrolyzing the peptide bond at the C‐terminus of any aromatic amino acid. Rennet, a protease not studied in Liang, was chosen initially for its familiarity to the dairy industry. The type of rennet used in this study is a fungal analog of calf chymosin. In contrast to Formea, this type of rennet is highly specific in its hydrolytic activity, primarily cleaving a single site on κ‐casein to release the caseinomacropeptide (Harboe, Broe, and Qvist 2010).

Enzyme was added to the WIC without adjusting pH or providing additional buffering. Although ice cream's typical pH (6.3, Goff and Hartel 2013b) and the incubation temperature (40°C) are not optimal for either enzyme, these conditions are within windows where both enzymes are expected to have substantial activity (Meng et al. 2018; Novozymes Switzerland 2017, 2019).

Although in preliminary experiments, WIC samples treated with Formea sometimes produced substantial demulsified fat, in the experiments presented here (Table 3), the amounts were too small to be reliably quantified or collected by our methods. The quantitation limit was defined as a free oil depth of 2 mm, which translates to a fat recovery of about 9%; at smaller depths, imperfections in phase interface made measurement and oil collection impractical.

**TABLE 3:** Fat recovery and quality using the experimental fat separation method and varying the enzyme type. All separations used pH adjustment to 9 and incubation at 50°C. Values are means of 3–4 replications ±1 standard deviation.

Variety ID	Enzyme type	Fat recovery	FFA^a^	PV^b^
		%	% as oleic acid	meq O_2_/kg
P V	Rennet	73.5 ± 2.3	1.9 ± 0.3	< 0.01
TH VB	Rennet	62.2 ± 3.1	1.5 ± 0.1	< 0.01
P V	Formea	< ql^c^	—	—
TH VB	Formea	< ql	—	—

^a^ Free fatty acids.

^b^ Peroxide value (milliequivalents O_2_/kg).

^c^ ql, quantitation limit.

Samples treated with rennet yielded substantial demulsified fat (Table 3), although certainly less than the standard fat recovery method (Table 2), and the recovery differed between varieties. While the FFA levels in the recovered fat were not particularly high, they were circa 30–40 times the corresponding levels found in the fat recovered by the standard method, indicating that hydrolysis is occurring during separation. A number of factors may have contributed to the elevated hydrolysis. Increased contact with water (Orthoefer and List 2007) and elevated temperature (Jadhav et al. 1995) are expected to have this effect. Lipases may also play a role; while endogenous milk lipases should be inactivated in pasteurization, bacterial lipases formed either pre‐ or postpasteurization may hydrolyze fat. Although some forms of rennet contain small amounts of lipase, the manufacturer claims that is not true of the product used in this study. While increased FFA is undesirable, this type of defect is correctable through refining (Farr 2014), and the context of this study is that the recovered fat would be refined regardless of the FFA content. Peroxide value results, on the other hand, were very low; in this respect, the experimental method appears to be superior to the standard method. These results, however, are not definitive of low oxidative damage; it is possible for highly oxidized oils to have a low PV due to further oxidation destroying the hydroperoxides.

The mechanism by which rennet promotes demulsification (and Formea mostly fails to) is unclear. In homogenized dairy products, the surface of fat globules contains milk proteins (along with remnants of the milk fat globule membrane and possibly emulsifiers) which sterically hinder globule coalescence. Hypothetically, the action of a nonspecific protease could release some fragments of these proteins into the aqueous phase and leave some fragments on the interface; the treated globule might be thus less resistant to coalescence. This may be occurring to some extent in Formea‐treated samples, but it does not seem to explain the much greater demulsification of the rennet‐treated samples. Rennet is not expected to cause a major reduction in the diameter of a casein micelle and seemingly would not have a major impact on steric hinderance (although electrostatic repulsion would be diminished due to the removal of the caseinomacropeptide). The rennet‐induced coagulation of casein micelles may play some role in demulsification, but this remains completely speculative. A study involving UHT milk (Zhang et al. 2020) demonstrated that the action of different proteases can result in either creaming or sedimentation of the fat, and showed the phenomena involved are more complex than envisioned in our digestion hypothesis.

### 3.4 Experimental Fat Separation Method—pH

This study was designed using an incorrect assumption that caseins are the predominant protein component of the material at the ice cream fat globule interface. Caseins normally exist in micelles and the size and structure of these micelles is pH‐sensitive (Sinaga, Bansal, and Bhandari 2017); relative to their state at milk's slightly acid pH (~6.6), increasing pH up to about 8.5 causes swelling of casein micelles; with further increases, they begin to dissociate. These considerations led to the hypothesis that pH greater than 8.5 would favor fat globule coalescence. Studies have shown, however, that β‐lactoglobulin is the predominant protein (Dalgleish, Goff, and Luan 2002).

**FIGURE 2:**
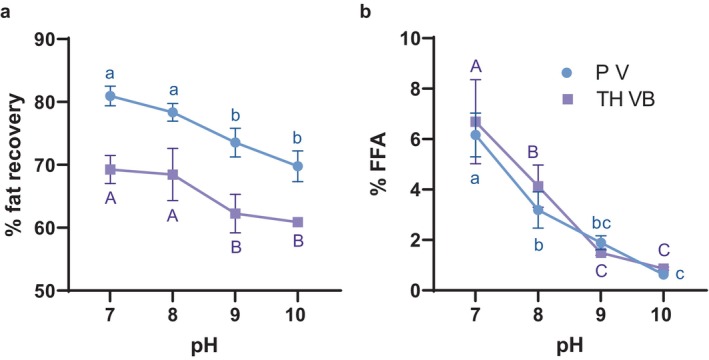
Percent of theoretical fat recovery (a) and percent free fatty acid content (b) when applying the experimental fat recovery method over a range of pH adjustment values. All separations used rennet and incubation at 50°C. Values are means of 3–4 replications ±1 standard deviation. Letter differences indicate statistically significant differences between means determined by two‐way ANOVA followed by Tukey's multiple comparison test (α = 0.05).

The results do not support the hypothesis (Figure 2a); rather, increases in pH tended to suppress recovery. The results may reflect the effect of pH on the structure of continuous phase proteins, and consequently, the viscosity of the continuous phase; increased viscosity tends to promote partial coalescence during the whipping and freezing of ice cream (Freire, Wu, and Hartel 2020). Viscosity was not measured in the present study.

The fat quality results complicate the selection of pH for the experimental fat separation method. While higher pH values were worse for fat recovery, they were better for preventing fat hydrolysis (Figure 2b). As discussed in the previous section, the primary mechanism of fat hydrolysis has not been identified, and pH‐sensitivity could be consistent with either chemical or enzymatic hydrolysis. Measured levels of oxidative damage were low, with most samples having PV below the limit of detection (data not shown); the only exceptions were 3 of 4 PV samples at pH 7—the most oxidized of these measured 0.08 meq O_2_/kg.

### 3.5 Experimental Fat Separation Method—Bath Temperature

**FIGURE 3:**
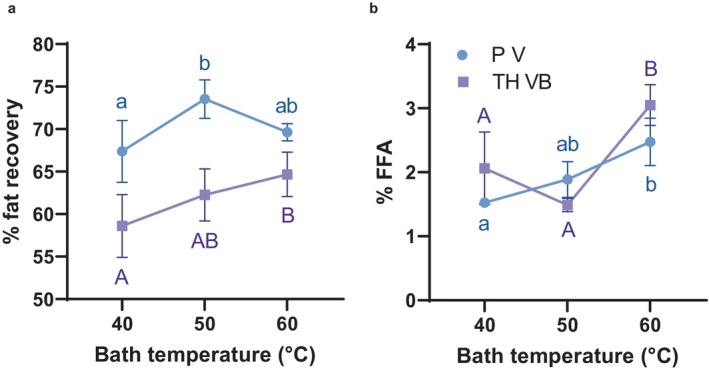
Percent of theoretical fat recovery (a) and percent free fatty acid content (b) when applying the experimental fat recovery method over a range of incubating bath temperatures. All separations used rennet, and pH adjustment to 9. Values are means of 3–4 replications ±1 standard deviation. Letter differences indicate statistically significant differences between means determined by two‐way ANOVA followed by Tukey's multiple comparison test (α = 0.05).

Incubating bath temperature was investigated as a factor that might influence fat recovery. Increased temperature sometimes promotes demulsification. Multiple factors favor the creaming of fat at elevated temperature—a greater density difference between butterfat and water, reduced viscosity of the aqueous phase, and collapse of partially coalesced fat globule clusters into lower‐drag spherical globules. On the other hand, it is known that globules that are too warm, and thus insufficiently crystallized, will resist coalescence (Fredrick, Walstra, and Dewettinck 2010). The experimental results were inconsistent (Figure 3a). Fat recovery from the variety TH VB improved with increased bath temperature, but P V recovery did not follow a clear trend. Fat hydrolysis was modestly higher for both varieties at 60°C compared to 40°C, and 1 of 4 samples of each variety gave PV greater than the detection limit (0.03 and 0.02 meq O_2_/kg for P V and TH VB, respectively).

### 3.6 Experimental Fat Separation Method—Skipping Steps

Studied as a whole, it is not clear whether the experimental fat separation method is excessively complex or whether each step is making a valuable contribution. To shed light on these topics, a series of separations were conducted in which one of the steps was omitted (Table 4). Every omission had a substantial negative impact, but the impacts varied greatly by omitted step. Skipping either the enzymatic digestion or the second centrifugation diminished the recovered fat yield below the level that could be quantified. Skipping the first centrifugation step was almost as impactful, except that with each WIC variety, 1 of the 4 replications gave measurable amounts of fat; the values reported for this treatment in Table 4 are the values of these single replications and are not means. The FFA values obtained from these replications were among the lowest from any variation of the experimental separation method reported in this study. Omission of the two remaining steps had lesser impact. Skipping pH adjustment reduced fat recovery, and gave very high FFA values, continuing a trend shown in Figure 2b. Skipping the hot bath incubation simply reduced fat recovery. Taken together, the results suggest no step in the experimental fat recovery method is superfluous.

**TABLE 4:** Fat recovery and quality using the experimental fat separation method with modifications to omit single steps. All separations used rennet, pH adjustment to 9, and incubation at 50°C. Values are means of four replications ±1 standard deviation.

Treatment	Variety ID	Fat recovery	FFA^a^	PV^b^
		%	% as oleic acid	meq O_2_/kg
All steps (no step omitted)	PV	73.5 ± 2.3	1.9 ± 0.3	< 0.01
	TH VB	62.2 ± 3.1	1.5 ± 0.1	< 0.01
Skip enzymatic digestion	PV	< ql^c^	—	—
	TH VB	< ql	—	—
Skip first centrifugation	PV ^4^	> 28.6	0.675	0.025
	TH VB^d^	> 7.6	0.755	< 0.01
Skip pH adjustment	PV	26.1 ± 3.5	11.7 ± < 0.1	0.07 ± 0.01
	TH VB	12.8 ± 3.7	11.9 ± 0.1	0.05 ± 0.01
Skip hot bath	PV	55.9 ± 4.5	1.3 ± 0.05	0.04 ± 0.01
	TH VB	44.4 ± 2.0	1.1 ± 0.28	< 0.01
Skip second centrifugation	PV	< ql	—	—
	TH VB	< ql	—	—

^a^ Free fatty acids.

^b^ Peroxide value (milliequivalents O_2_/kg).

^c^ ql, quantitation limit.

^d^ Anomalous results; refer to text.

### 3.7 Experimental Fat Separation Method—General Applicability

The superficial similarities of vanilla ice cream products obscure diversity in formulation and processing of these products. To evaluate the applicability of the experimental fat separation method, it was tested on the same set of five products used with the standard fat separation method (Section 3.2). The fat recovery varied widely, but with four of the varieties, more than half of the fat was recovered, the FFA levels were moderate, and the PV was too low to measure. No recoverable fat was observed with the remaining variety; the data do not provide any explanation for the anomalous behavior of this variety, but it was only variety that was formulated with both mono‐ and diglycerides and egg yolk as emulsifiers (See Tables 1 and 5).

**TABLE 5:** Fat recovery and quality using the experimental fat separation method on a broad range of varieties. All separations used rennet, pH adjustment to 9, and incubation at 60°C. Values are means of four replications ±1 standard deviation.

Variety ID	Fat recovery	FFA^a^	PV^b^
	%	% as oleic acid	meq O_2_/kg
P V	69.6 ± 1.0	2.5 ± 0.4	< 0.01
TH VB	64.7 ± 2.6	3.1 ± 0.3	< 0.01
B NV	71.8 ± 3.1	2.4 ± 0.0	< 0.01
TH FB	< ql^c^	—	—
HB V	53.5 ± 6.3	1.1 ± 0.0	< 0.01

^a^ Free fatty acids.

^b^ Peroxide value (milliequivalents O_2_/kg).

^c^ ql, quantitation limit.

## 4 Conclusions

A multistep demulsification process is capable of recovering the majority of fat from most varieties of WIC. This process avoids the large reagent consumption and waste generation of the standard solvent extraction method but sacrifices both fat yield and quality.

Further development of this process would be facilitated by a better understanding of the mechanism by which rennet promotes demulsification. Other questions remain which will impact the practicality of this process including the fate of the byproduct stream, which may be useful in livestock feed or fermentation systems, and the presence of emulsifiers in the recovered fat and whether these can be eliminated through refining. Cost modeling of a hypothetical industrial implementation of this process could be used to direct development toward improved viability.

## Author Contributions

**Rafael A. Garcia:** conceptualization (equal), data curation (lead), formal analysis (lead), methodology (equal), project administration (lead), resources (lead), supervision (lead), visualization (lead), writing – original draft (lead). **Chen Liang:** conceptualization (equal), investigation (equal), methodology (equal), writing – review and editing (equal). **Benjamin M. Plumier:** investigation (equal), methodology (supporting), writing – review and editing (equal). **Changhoon Lee:** investigation (equal), methodology (equal), writing – review and editing (equal). **Lorelie P. Bumanlag:** investigation (equal), methodology (equal), writing – review and editing (equal). **John A. Renye:** writing – review and editing (equal). **Peggy M. Tomasula:** writing – review and editing (equal).

## Conflicts of Interest

The authors declare no conflicts of interest.

## Data Availability

The data that support the findings of this study are available on request from the corresponding author.
